# Supplementary material for: MetaRibo-Seq measures translation in microbiomes
Source: Nat Commun. 2020 Jun 29;11:3268. doi: 10.1038/s41467-020-17081-z (PMC7324362; doi:10.1038/s41467-020-17081-z)
Supplement: Supplementary file 10 — Supplementary Data 7 [file 41467_2020_17081_MOESM10_ESM.zip › File2/Confidence_High_Taxonomy/357868_out.krona.html]

Javascript must be enabled to view this page.

members
magnitude
magnitudeUnassigned
count
unassigned
taxon
rank

357868\_out

5

2
superkingdom
5

5
976
phylum

class
200643
5

171549
order
5

family
171552
5

838
genus
5

5

SRS013521\_contig\_number\_contig-100\_860.860SRS017307\_contig\_number\_14796SRS054059\_contig\_number\_117SRS1041031\_contig\_number\_contig-100\_176.160690SRS1041140\_contig\_number\_21443
species
165179
